# Evaluation of the Genotoxic and Antigenotoxic Effects of Chios Mastic Water by the *In Vitro* Micronucleus Test on Human Lymphocytes and the *In Vivo* Wing Somatic Test on *Drosophila*

**DOI:** 10.1371/journal.pone.0069494

**Published:** 2013-07-23

**Authors:** Dimitris Vlastos, Despoina Mademtzoglou, Elena Drosopoulou, Ioanna Efthimiou, Tatiana Chartomatsidou, Christina Pandelidou, Melina Astyrakaki, Eleftheria Chalatsi, Penelope Mavragani-Tsipidou

**Affiliations:** 1 Department of Genetics, Development and Molecular Biology, Aristotle University of Thessaloniki, Thessaloniki, Greece; 2 Department of Environmental and Natural Resources Management, University of Patras, Agrinio, Greece; University of Crete, Greece

## Abstract

Chios mastic gum, a plant-derived product obtained by the Mediterranean bush *Pistacia lentiscus* (L.) var. *chia* (Duham), has generated considerable interest because of its antimicrobial, anticancer, antioxidant and other beneficial properties. Its aqueous extract, called Chios mastic water (CMW), contains the authentic mastic scent and all the water soluble components of mastic. In the present study, the potential genotoxic activity of CMW, as well as its antigenotoxic properties against the mutagenic agent mitomycin-C (MMC), was evaluated by employing the *in vitro* Cytokinesis Block MicroNucleus (CBMN) assay and the *in vivo* Somatic Mutation And Recombination Test (SMART). In the former assay, lymphocytes were treated with 1, 2 and 5% (v/v) of CMW with or without MMC at concentrations 0.05 and 0.50 µg/ml. No significant micronucleus induction was observed by CMW, while co-treatment with MMC led to a decrease of the MMC-induced micronuclei, which ranged between 22.8 and 44.7%. For SMART, larvae were treated with 50 and 100% (v/v) CMW with or without MMC at concentrations 1.00, 2.50 and 5.00 µg/ml. It was shown that CMW alone did not modify the spontaneous frequencies of spots indicating lack of genotoxic activity. Τhe simultaneous administration of MMC with 100% CMW led to considerable alterations of the frequencies of MMC-induced wing spots with the total mutant clones showing reduction between 53.5 and 74.4%. Our data clearly show a protective role of CMW against the MMC-induced genotoxicity and further research on the beneficial properties of this product is suggested.

## Introduction

*Pistacia lentiscus* (L.) var. *chia* (Duham) is an evergreen bush, uniquely cultivated in the Greek island Chios [1]–[3]. It produces a white semitransparent resin, which is generally known as Chios mastic gum. This product as well as its essential oil, Chios mastic oil, has been extensively used as food/beverages flavoring additives in confectionery, in perfume industry and as an ingredient of cosmetics and health products [3]–[5]. Their beneficial biological properties have been well documented by a number of studies showing their antibacterial, antimicrobial, anti-inflammatory and antioxidant activity [5]–[13] and they have been proposed for many clinical applications [14]–[21]. Recently, their anticancer properties against a number of human malignancies have been reported [22]–[28]. Despite the great number of reports analyzing the biological activities of mastic gum and mastic oil, such studies are scarce for the very closely related commercially available product, known as Chios mastic water (CMW).

CMW is a flavoring obtained in large quantities together with mastic oil during the steam distillation of mastic resin. It is a 100% natural aqueous extract that contains all the water soluble components of mastic gum as well as a small amount (0.5–1% v/v) of mastic oil [data from Chios Mastiha Growers’ Association, CMGA]. Its major identified compounds are verbenone, α-terpineol, trans-p-menth-2-ene-1,8-diol, cis-p-menth-2-ene-1,8-diol, linalool, β-phellandrenol and trans-pinocarveol [29]. With the exception of a recent study on its chemical composition and its antimicrobial activities against *Escherichia coli*, *Staphylococcus aureus* and *Candica* spp. [29], data on the biological properties of this low-cost product of mastic resin do not exist.

In an effort to evaluate the safety of use of CMW, the possible genotoxic and recombinogenic effects of this mastic product were studied here. To further explore its biological properties, the potential protective effects of CMW against the mutagenic and recombinogenic effects of mitomycin-C (MMC) were also investigated. Both genotoxic and antigenotoxic potential activities of CMW were assessed employing the cytokinesis block micronucleus (CBMN) assay and the somatic mutation and recombination test (SMART). The former is an *in vitro* assay applied in cultured human lymphocytes for the detection of micronuclei (MN) in the cytoplasm of interphase cells. MN may originate from acentric chromosome fragments or whole chromosomes that are unable to migrate to the poles during the anaphase stage of cell division. Thus, this assay detects the potential clastogenic and aneugenic activity of chemicals in cells that have undergone cell division after exposure to the test chemical [30], [31]. The simplicity, rapidity and sensitivity of the CBMN assay make it a valuable tool for genotoxicity screening. Moreover, the SMART test in *Drosophila melanogaster* (Meigen) used here, is a sensitive, low-cost, rapid eukaryotic *in vivo* assay able to detect the potential mutagenic and recombinogenic effects as well as the antigenotoxic ability of chemicals. Thus, a wide spectrum of genetic end points such as point mutations, deletions, certain types of chromosome aberrations, as well as mitotic recombination and gene conversion can be detected [32], [33]. The extensive knowledge on the genetics of *D. melanogaster* and the high homology between fly and human genes [34]–[38] have made this organism unique in mutation research and genetic toxicology.

Since MN formation and recombinogenic events are found to be associated with carcinogenesis [39], [40], our results are expected to contribute to the establishment of the safety status of this commercially available mastic product. Moreover, its potential antigenotoxic activity against mutagens could contribute to the development of chemopreventive agents capable of modulating the cellular responses to mutagens (or of phytopharmaceutical molecules of interest).

## Materials and Methods

### Chemicals

The CMW was supplied by CMGA (Chios, Greece). MMC and cytochalasin-B (Cyt-B) were purchased from Sigma (St. Louis, MO, USA). Ham’s F-10 medium, foetal bovine serum and phytohaemaglutinin were commercially supplied (Gibco, UK). Faure’s solution was prepared by mixing 100 g distilled water (H_2_O), 100 g chloral hydrate (C_2_HCl_3_O.H_2_O), 40 g glycerine (C_3_H_8_O_3_) and 60 g arabic gum. All other chemicals and solvents were of the highest grade commercially available. Stocks of the compounds and solutions were stored at 4°C until use.

### Ethics Statement

The study was approved by the Ethical Committee of the University of Patras. After informed consent two healthy, non-smoking male individuals (less than 30 years), were used as blood donors to establish whole blood lymphocyte cultures. According to the donors’ declaration, they were not exposed to radiation, drug treatment or any viral infection in the recent past.

### CBMN Assay in Human Lymphocytes *in vitro*

Blood samples were kept under sterile conditions in heparinized tubes. Whole blood (0.5 ml) was added to 6.5 ml Ham’s F-10 medium, 1.5 ml foetal bovine serum and 0.3 ml phytohaemaglutinin to stimulate cell division.

CMW was added to final concentrations of 1, 2 and 5% (v/v) in culture volume either alone or in combination with 0.05 and 0.50 µg/ml of MMC. The MMC concentrations used in the present study have been previously used as positive control in the particular assay and cell type [41]. The appropriate volumes were added 24 h after culture initiation. Cyt-B at final concentration of 6 µg/ml was added to the culture medium 44 h after its initiation and 20 h after the addition of the CMW, MMC or their mixtures. This concentration of Cyt-B was selected in order to obtain a higher percentage of binucleated (BN) cells and a lower baseline MN frequency [42]. Cultures were incubated at 37°C in a humidified atmosphere of 5% CO_2_ for 72 h. 72 h after the initiation of culture, cells were harvested and collected by centrifugation. A mild hypotonic treatment with 3∶1 solution of Ham’s medium and milli-q H_2_O was left for 3 min at room temperature which was followed by 10 min fixation (for at least 3 times) with a fresh 5∶1 solution of methanol/acetic acid. Cells were stained with 7% Giemsa [43]–[45].

In total, 2000 BN cells with preserved cytoplasm were scored per experimental point. Standard criteria were used for scoring MN [46], [47] and the scoring of micronuclei was performed manually and by (at least) two, independently working, experienced researchers. In order to determine possible cytotoxic effects, the cytokinesis block proliferation index (CBPI) was calculated by counting at least 1000 cells for each experimental point (500 cells per culture of each donor). CBPI is given by the equation: CBPI = M_1_+2M_2_+3(M_3_+M_4_)/N where M_1_, M_2_, M_3_ and M_4_ correspond to the numbers of cells with one, two, three and four nuclei and N is the total number of cells [48].

### Somatic Mutation and Recombination Test (SMART)

Two *D. melanogaster* strains carrying visible wing genetic markers on the left arm of the third chromosome were used: (i) flare (*flr^3^*, 3–38.8) with genetic constitution *ywco/y wco; flr3 se/TM2 Ubx^130^ se e* and (ii) multiple wing hairs (*mwh*, 3–0.3) with genetic constitution *fs(1)K10 w/Y;mwh se e/mwh se e* [49], [50]. More detailed information on the genetic symbols and descriptions is provided by Lindsley and Zimm [49]. Insects were maintained at 24±1°C, at a photoperiod 16∶8 (light:dark) on a yeast–glucose medium. The experiments were carried out following the principles and the basic procedures presented by Graf et al. [32], [33]. Thus, eggs obtained by parental crosses between *flr^3^* virgin females and *mwh* males were collected during a 6-hour period in culture bottles with an agar-agar base (4% w/v) topped with a thick layer of live yeast supplemented with sucrose. Three days after egg laying, larvae in the third stage of embryonic development were washed out of the bottles with Ringer’s solution and collected in a stainless steel strainer. Series of 30 larvae were transferred for chronic feeding to treatment vials containing 0.85 g of *Drosophila* Instant Medium (Carolina Biological Supply, Burlington, NC, USA) rehydrated with 4 ml of 50 and 100% (v/v) CMW alone or in combination with MMC at final concentrations of 1.00, 2.50 and 5.00 µg/ml. The above concentrations of MMC were also used as positive control. Larvae were fed on these culture media for the rest of their larval life (approximately 48 h). The hatched adult flies were collected from the treatment vials and stored in 70% v/v ethanol/glycerol (1∶1, v/v). The wings of the trans-heterozygous (*mwh flr*+/*mwh*+*flr^3^*) female flies [32], [50], [51], distinguished by their wild-type body color, were removed under a stereomicroscope with a pair of entomological tweezers, mounted in Faure’s solution and scored at 400× magnification for the presence of mosaic spots. The rest individuals were excluded from analysis, because in the *mwh/TM2* females and the males recombinational events are suppressed [32], [50], [51]. The spots observed on the wings of the trans-heterozygous females were grouped into four categories based on the size, number and type of cells showing malformed wing hairs as: (i) small single spots (with one or two affected cells, either *mwh* or *flr^3^*), (ii) large single spots (with three or more affected cells, either *mwh* or *flr^3^*), (iii) twin spots (consisting of both *mwh* and *flr^3^*subclones), and (iv) total spots [32]. Single spots (*mwh* or *flr^3^*) are produced by various genetic events including somatic point mutations, deletions and other types of structural rearrangements as well as by mitotic recombination between the two marker genes, while twin spots (*mwh* and *flr^3^*) are produced exclusively by mitotic recombination occurring between the proximal marker *flr^3^* and the chromosome 3 centromere [32]. For comparative analysis, parallel experiments using distilled water were carried out as the negative controls. Ten replicates per treatment were performed. Since no considerable difference in survival rates of hatched flies from independent experiments was observed, approx. 50 wing samples per treatment were randomly selected for genotoxic analysis. All experiments were performed at 24±1°C and 60% RH. A total of about 600 wings were scored in this study.

### Statistical Analysis

All results of the CBMN assay are expressed as the mean frequency ± standard error (MF ± se). The G-test for independence on 2×2 tables was used to perform the statistical analysis of the MN data. The chi-square test (*χ^2^* test) was used for the analysis of CBPI among each treatment. Differences at p<0.05 were considered significant. The statistical software used for data analysis was the Origin 7.0 (OriginLab Corporation, Northampton, MA, USA), the Minitab statistical software (Minitab Inc., PA, USA) and the Statistical Package for Social Sciences (SPSS) for Windows, version 17.0.

Statistical analysis of the data derived by the SMART assay was done using the multiple-decision procedure [52], [53] which is based on the conditional binomial test and the chi-squared test (K. Pearson’s criterion) [54], [55]. A significance level of 5% was used. For the statistical assessment of antigenotoxicity, the frequencies of each type of spots per fly were compared in pairs (negative control versus CMW; MMC versus MMC+CMW), using the non-parametric Mann-Whitney U-test [56]. Based on clone formation per 10^5^ cells the percentages of CMW inhibition were calculated as follows: [(MMC - MMC combined with CMW)/MMC] x 100 [57].

## Results

### Genotoxicity and Antigenotoxicity Tested with CBMN Assay

Chios mastic water was studied for genotoxicity at three different doses i.e. 1, 2 and 5% (v/v) of the total culture volume and the same doses were tested combined with different MMC concentrations (0.05 and 0.50 µg/ml) in order to identify the antigenotoxic effect of CMW against the genotoxic damage induced by MMC. A treatment with 1, 2 and 5% (v/v) of CMW doses did not induce MN at significant level as compared to control. Treatments with 0.05 and 0.50 µg/ml of MMC induced significant MN frequencies (57.0±6.0 and 177.5±16.5) as compared to control. A significant decrease in MN frequencies was observed when 1, 2 and 5% (v/v) of CMW treatments were given along with both tested concentrations of MMC (Table 1). To summarize, the concentrations of CMW used in the present study were not genotoxic themselves, while they reduced the genotoxic effect of MMC.

**Table 1:** Frequencies of BNMN and MN as well as CBPI values in cultured human lymphocytes treated with CMW, MMC (0.05 and 0.50 µg/ml) and their mixture.

Treatment	BNMN MF (‰) ± se	MN MF (‰) ± se	CBPI MF (‰) ± se
Control	4.5±0.5	5.0±1.0	1.89±0.04
1% (v/v) CMW	5.0±2.0	5.5±2.5	1.87±0.04
2% (v/v) CMW	4.5±0.5	4.5±0.5	1.74±0.04^2^
5% (v/v) CMW	9.0±1.0	9.0±1.0	1.64±0.03^3^
MMC (0.05 µg/ml)	55.5±6.5 ^3^	57.0±6.0^3^	1.67±0.02^3^
1% (v/v) CMW+MMC (0.05 µg/ml)	30.5±0.5 ^3,c^	31.5±0.5^3,c^	1.69±0.02^3,a^
2% (v/v) CMW+MMC (0.05 µg/ml)	32.5±2.5 ^3,c^	34.0±2.0^3,c^	1.76±0.02^1,a^
5% (v/v) CMW+MMC (0.05 µg/ml)	36.0±2.0 ^3,b^	36.5±2.5^3,b^	1.68±0.05^3,a^
MMC (0.50 µg/ml)	166.0±15.0^3^	177.5±16.5^3^	1.50±0.04^3^
1% (v/v) CMW+MMC (0.50 µg/ml)	126.5±1.5^3,c^	137.0±1.0^3,c^	1.48±0.00^3^
2% (v/v) CMW+MMC (0.50 µg/ml)	128.5±2.5^3,c^	134.5±1.5^3,c^	1.50±0.03^3^
5% (v/v) CMW+MMC (0.50 µg/ml)	128.5±26.5^3,c^	134.5±29.5^3,c^	1.40±0.01^3,c^

BN: binucleated cells; BNMN: micronucleated binucleated cells; MN: micronuclei; CBPI: Cytokinesis Block Proliferation Index; CMW: Chios Mastic Water; MMC: Mitomycin-C; MF (‰) ± se, mean frequencies (‰) ± standard error; MN were scored in 2000 binucleated lymphocytes per experimental point;

1,2,3 significant difference in relation to control at *p*<0.05, *p*<0.01 and *p*<0.001 respectively;

a,b,c significant difference in relation to MMC at *p*<0.05, *p*<0.01 and *p*<0.001 respectively [G-test for BNMN and MN; *χ2* for CBPI].

**Figure 1:**
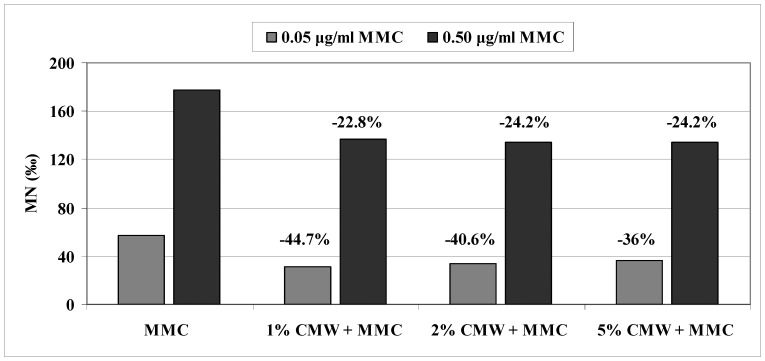
Reduction (%) of MN frequency induced by MMC (0.05 and 0.50 µg/ml) in presence of CMW (1, 2 and 5% v/v).

**Figure 2:**
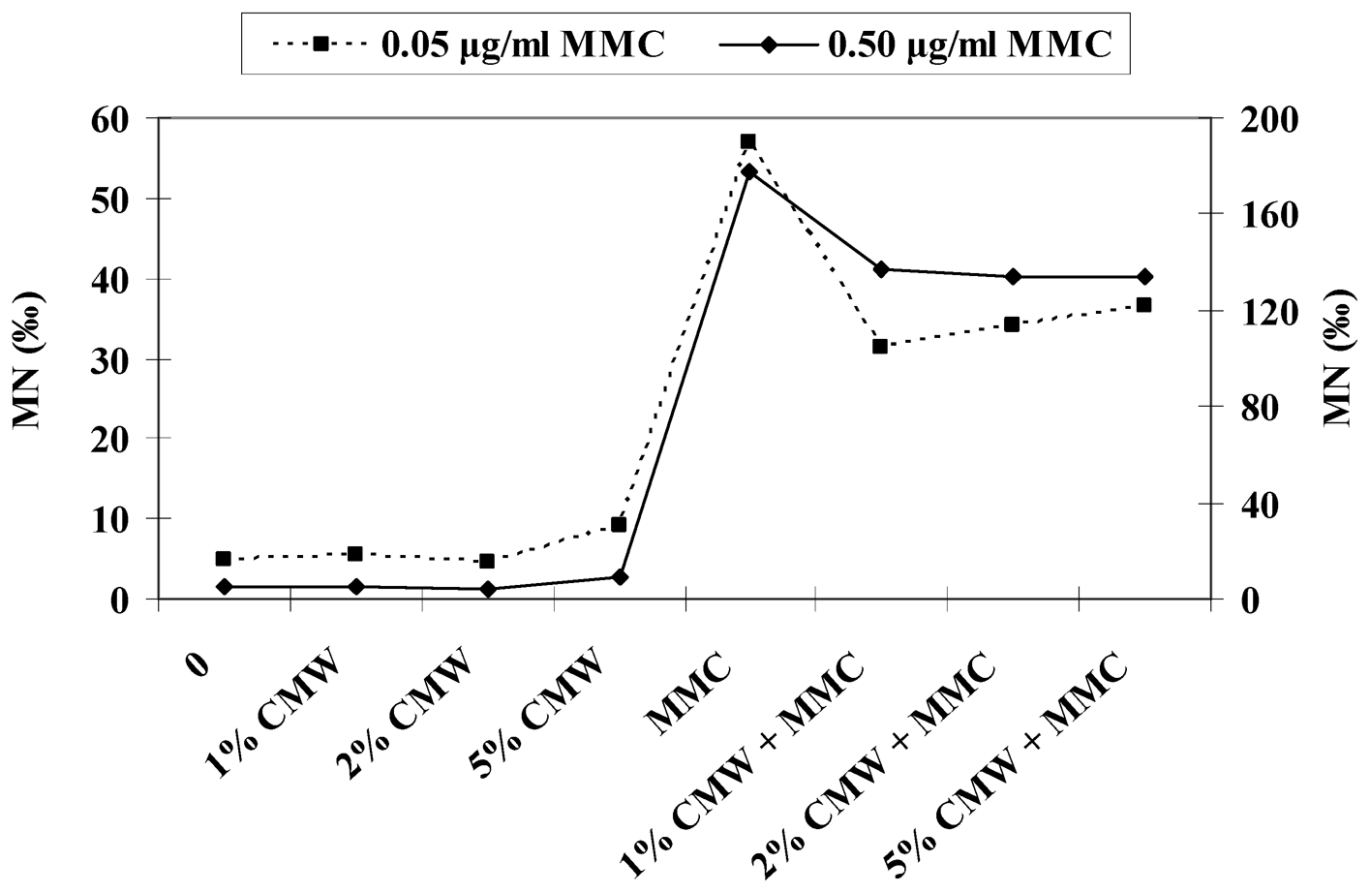
Comparative distribution of MN frequency (‰) induced by CMW, MMC and their combination. The dotted line is read on the left hand Y-axis and the solid line on the right hand Y-axis.

Figure 1 shows the reduction of MMC-induced MN frequencies (%) in the presence of different concentrations of CMW. In the tested concentrations of MMC (0.05 and 0.50 µg/ml) the decrease of the MN frequencies ranges from 22.8 to 44.7%. The comparative distribution of MN frequency induced by CMW, MMC and their combination is indicated in Figure 2. A similar pattern is shown in both MMC concentrations. In particular, a decrease in MN frequency induction is observed in co-treatment with CMW and MMC in comparison to MMC alone in both concentrations, with slightly greater decrease of the induction frequency in the CMW and 0.05 µg/ml MMC mixtures.

The cytotoxic effect of CMW, MMC and their mixtures was evaluated by the determination of CBPI. Regarding the cytotoxic index, statistically significant differences on CBPI were detected between control cultures and the 2 and 5% (v/v) doses of CMW. The decrease of the CBPI index with some fluctuations remains in the case of MMC as well as in the mixtures of CMW with MMC (Table 1).

### Genotoxicity and Antigenotoxicity Tested with SMART Assay

In a pilot experiment, the toxicity of CMW was evaluated. No toxicity of this product could be determined in *Drosophila* (data not shown). Therefore, CMW was applied at concentrations 50 and 100% (v/v) for the genotoxicity experiments. MMC was supplied to *D. melanogaster* larvae at doses of 1.00, 2.50 and 5.00 µg/ml, since preliminary experiments showed that lower concentrations did not exert strong genotoxic effects under our experimental conditions (data not shown). The antigenotoxic effect of CMW against the genotoxic damage induced by MMC was accomplished by co-treatment of the above doses of both compounds.

Table 2 summarizes the results together with the negative control experiment. No significant differences in any of the three spot categories were observed after chronic treatment of *Drosophila* larvae with CMW, compared to those of their respective negative controls, indicating that CMW was not genotoxic under our experimental conditions. On the other hand, treatment of the larvae with MMC at concentrations 1.00, 2.50 and 5.00 µg/ml evoked a statistically significant rise in all spot categories. The correlation between the dose and the frequency of the induced total spots indicates the dose dependent genotoxic activity of MMC. Moreover, the positive effect of twin spots at the high concentrations used clearly indicates the recombinogenic activity of this agent.

**Table 2:** Summary of the results obtained in the Somatic Mutation And Recombination Test (SMART) on *Drosophila melanogaster,* after larvae treatment with CMW, MMC (1.00, 2.50 and 5.00 µg/ml) and their mixture.

Treatment	Number of wings	Frequency of spots per wing and diagnosis^1^			
		Small single spots *m* = 2.0	Large single spots *m* = 5.0	Twin spots*m* = 5.0	Total spots *m* = 2.0
Control	50	0.460 (23)	0.040 (2)	0.020 (1)	0.520 (26)
50% (v/v) CMW	50	0.460 (23) −	0.000 (0) −	0.100 (5) i	0.560 (28) −
100% (v/v) CMW	50	0.320 (16) −	0.020 (1) i	0.020 (1) i	0.360 (18) −
MMC (1.00 µg/ml)	47	0.745 (35) +	0.234 (11) +	0.043 (2) i	1.021 (48) +
50% (v/v) CMW+MMC (1.00 µg/ml)	46	0.500 (23) −	0.152 (7) +	0.022 (1) i	0.740 (31) i
100% (v/v) CMW+MMC (1.00 µg/ml)	46	0.348 (16) −^a^	0.087 (4) i	0.000 (0) i	0.435 (20) −^a^
MMC (2.50 µg/ml)	50	1.540 (77) +	0.720 (36) +	0.400 (20) +	2.660 (133) +
50% (v/v) CMW+MMC (2.50 µg/ml)	48	1.021 (49) +	0.667 (32) +	0.333 (16) +	2.021 (97) +
100% (v/v) CMW+MMC (2.50 µg/ml)	47	0.426 (20) −^b^	0.255 (12)+^a^	0.000 (0) i^c^	0.681 (32) i^c^
MMC (5.00 µg/ml)	50	2.580 (129) +	2.040 (102) +	0.540 (27) +	5.160 (258) +
50% (v/v) CMW+MMC (5.00 µg/ml)	49	2.102 (103) +	1.612 (79) +	0.429 (21) +	4.143 (203) +
100% (v/v) CMW+MMC (5.00 µg/ml)	50	1.200 (60)+^b^	0.880 (44)+^b^	0.320 (16)+^b^	2.400 (120)+^c^

Symbols next to values signify the following: +, positive mutagenic effect; −, no mutagenic effect; w, weakly positive effect; i, inconclusive effect; *m* is the multiplication factor^1^ (*p = *0.05); ^a, b, c^ is significant difference in relation to MMC at *p*<0.05, *p*<0.01 and *p*<0.001, respectively (U-test).

1 The number of mutant spots is given in parenthesis. Statistical diagnosis according to Frei and Würgler [53].

**Figure 3:**
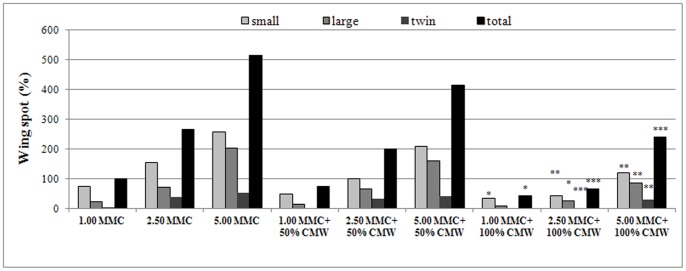
Wing spot frequency (%) induced by MMC (1.00, 2.50 and 5.00 µg/ml) in presence of CMW (50 and 100% v/v). [*p<0.05, **p<0.01, ***p<0.001, U-test].

After co-treatment of MMC with 50% (v/v) CMW, a reduction of the induced total wing spot frequency was observed, which, however, was not found to be statistically significant (*U* = 238.5, *p* = 0.296, *U* = 230.0, *p* = 0.158, *U* = 210.0, *p* = 0.071, for 1.00, 2.50 and 5.00 µg/ml MMC, respectively) (Figure 3). On the contrary, a more pronounced decrease of MMC-induced total spots was provoked by 100% (v/v) CMW. This overall inhibition was 57.4%, (*U* = 189.0, *p* = 0.032), 74.4% (*U* = 92.5, *p* = 0.000) and 53.5% (*U* = 129.0, *p* = 0.000) in the case of 1.00, 2.50 and 5.00 µg/ml MMC, respectively (Figure 3). It should be noted that when 100% (v/v) CMW is supplied along with 1.00 and 2.50 µg/ml MMC, the frequencies of total spots are similar to those observed in the negative control meaning that CMW is able to inhibit completely the genotoxic activity of MMC at these concentrations (Table 2). On the other hand, when 100% (v/v) CMW is combined with 5 µg/ml MMC, even though the reduction of total spots is over 50% and statistically significant (*U* = 129.0, *p* = 0.000), the genotoxic result remains positive. Considering spot sub-categories, 100% (v/v) CMW reduced significantly small single and total spots induced by 1.00 µg/ml MMC (*U* = 193.0, p = 0.037) and all spot categories induced by 2.50 and 5.00 µg/ml MMC (*U* = 115.0–191.0, p = 0.000–0.031) indicating both antigenotoxic and antirecombinogenic activity.

## Discussion

In recent years, mastic gum, a natural resin obtained from the plant *Pistacia lentiscus* var. *chia*, its essential oil (i.e. mastic oil), as well as some of their constituents [e.g. linalool, verbenone, α-terpineol, *trans-*pinocarveol] have received much attention as potentially useful bioactive compounds, with particular emphasis being given to their antioxidant, antimicrobial, anti-inflammatory or antitumor properties [5]–[29], [58]–[65]. Based on the increasing international interest for mastic products, in the present study, the CMW was evaluated, for the first time, for its potential genotoxic effect as well as its antigenotoxic activity against the DNA damage induced by MMC.

For this purpose, the *in vitro* CBMN assay in cultured human lymphocytes and the *in vivo* SMART test in *D. melanogaster* were applied. Both assays are short-term genotoxicity tests able to evaluate several genetic endpoints during the cell cycle or special developmental stages [30]–[32].

In our testing systems, CMW was not found to be genotoxic, mutagenic or recombinogenic, as it did not induce increased frequencies of micronuclei or wing spots within a wide range of concentrations (Tables 1–2). To our knowledge there is no data on the genotoxic activity of CMW; nevertheless, one of its major constituents, linalool, with contribution 7.29% [29] was found not to exhibit genotoxic or recombinogenic activity [65]. Moreover, borneol which has low contribution (0.99%) in CMW [29] has been reported not to be genotoxic at low concentrations [61]. Since the organic fraction of CMW is a complex mixture of many constituents [29], the absence of genotoxicity found in the present study could be attributed either to its major constituents or to synergistic and/or antagonistic phenomena that may exist among its constituents [66], [67]. Our results are also in line with previous reports showing that extracts of *P. lentiscus* did not exert any genotoxic effects [68], [69].

As it can be seen in Table 1, a significant decrease of CBPI values were noticed at concentrations 2 and 5% (v/v) of CMW. The above observation is related with the CMW constituents and is supported by literature data demonstrating that some of these constituents, namely verbenone, α-terpineol, linallol, α-phellandrenol, myrtenol/myrtenal, terpinen-4-ol and borneol, have been shown to exhibit cytotoxic activity [59]–[61], [63], [64].

Since no genotoxic activity was detected at any concentration tested, the potential antigenotoxic activity of CMW was evaluated, herein. For this purpose, MMC was used as a mutagenic inducer, similarly to a number of other antigenotoxicity studies [70]–[74]. MMC is an alkylating, antibiotic compound that has a range of genotoxic effects including the inhibition of DNA synthesis, mutagenesis and clastogenesis. It was found to be genotoxic in all *in vitro* and *in vivo* test systems in mammalian cells and animals and was clearly demonstrated as carcinogenic agent [75]. Consistent with previous studies [70]–[77], MMC was found to be genotoxic in both our testing assays, inducing significant MN and wing spot frequencies at concentrations 0.05 and 0.50 µg/ml in the CBMN assay and over 1.00 µg/ml in the SMART test (Tables 1, 2). In addition, the significant induction of twin spots in SMART test indicated the recombinogenic activity of this agent (Table 2).

The co-treatment of human lymphocytes and *D. melanogaster* larvae with CMW and MMC demonstrated that CMW could afford protection against the used mutagen indicating its antigenotoxic activity under both our *in vitro* and *in vivo* testing conditions. More precisely, the frequency of micronuclei and wing spots was statistically decreased when MMC was combined with CMW in comparison to the micronuclei and spot frequencies induced by MMC alone (Tables 1–2, Figures 1, 2, 3). In the CBMN assay, the decrease of the frequency of MN induction ranged between 36.0% and 44.7% and between 22.8% and 24.2% for 0.05 and 0.50 µg/ml of MMC, respectively. The antigenotoxic capacity of CMW was further supported by the results of the SMART test, which showed a profound reduction of total mutant spots on the wing blade after co-treatment with MMC and 100% (v/v) CMW compared to MMC alone. The decrease of the total spots was found to be 57.4, 74.4 and 53.5% for 1.00, 2.50 and 5.00 µg/ml of MMC, respectively. The significant reduction of the frequencies of single (small and large) and twin spots indicate that 100% (v/v) CMW has a protective effect against the MMC’s genotoxic and recombinogenic action (Table 2, Figure 3).

It is of note that in the CBMN assay the most profound decrease of MMC-induced MN (i.e. 44.7%, Figure 1) was observed when the lowest examined CMW concentration (i.e. 1% v/v) was co-administered to 0.05 µg/ml of MMC. These results are in accordance with those of Kim and Neophytou [62], who observed that the lowest dose of mastic oil (0.02% v/v) had a stronger influence than the highest one (0.2% v/v) with respect to the decrease of the symptoms of clinical colitis in mice. Moreover, recent studies reported that beneficial effects of mastic gum/extracts could be achieved at low doses [22]–[24], [26], [58]. These findings lead to a possible assumption that low concentrations of mastic’s drastic constituents display the desirable effects in both antigenotoxic and therapeutic levels. The above is corroborated by a study of Doi et al. [78], according to which Chios mastic gum at high doses enhances the induction of preneoplastic lesions in rat liver. Thus, as proposed for other substances [79]–[82], some of CMW’s constituents could potentially act as free radical scavengers at low concentrations and as pro-oxidants at higher concentrations. However, in our testing systems, the antigenotoxic activity of CMW was accomplished by quite different concentrations. Thus, even though in the CBMN assay CMW could afford protection against the used mutagen at low concentrations (1, 2 and 5% v/v), in the SMART test this was obtained by 100% (v/v) CMW (Table 2). In the latter assay 50% (v/v) CMW was unable to significantly reduce the MMC-induced mutagenic effects while 100% (v/v) CMW was capable to abolish completely the mutagenic effects induced by low concentrations of MMC (1.00 and 2.50 µg/ml) (Table 2). A number of factors may influence the observed differences in the *in vivo* and *in vitro* assays, such as compound absorption, rate and distribution of biotransportation, availability at the target site and cell permeability [83].

The antigenotoxic effects of CMW found, here, are supported by literature data demonstrating that some of its constituents, namely linalool [84], borneol [61] and perillyl alcohol [85] have been shown to exhibit antigenotoxic activity. Despite the low content of CMW in borneol and perillyl-alcohol, linalool is detected in a percentage of 7.29%, constituting one of CMW’s major compounds [29]. Nevertheless, it should not be overseen that the observed protective effects of CMW would most likely be attributed to the additive/synergistic interaction of many major or minor constituents or to the combination of more than one biological activities [26], [69]. Our data are consistent with the previously reported antigenotoxic and antioxidant properties of *P. lentiscus* extracts [68], [69] and the antioxidant properties [5], [9], [12] as well as the anticancer effects of Chios mastic gum against a number of malignancies [22]–[28].

MMC is used in clinical cancer chemotherapy against a variety of solid neoplasms. However, due to its mutagenic and/or carcinogenic ability, secondary cancers are generated, which became a serious problem of chemotherapy. Thus, identifying new non-toxic phytochemicals capable of preventing DNA damage of MMC is very important in developing novel nutraceuticals. Our results clearly show that CMW prevents or reduces DNA damage induced by MMC. Even though the mechanism of interaction between MMC and CMW is not known, the co-treatment protocol, used in the present study, cannot rule out the possibility of CMW acting as a desmutagen and interacting with the active groups of MMC [86]. Since CMW was present at the time of MMC exposure, it could inhibit the cytosol flavoreductases that activate MMC [87] and, thus, could block its activation and the subsequent DNA damage [88]. On the other hand, the observed antigenotoxic activity of CMW could also be ascribed to the mastic’s antioxidant effects [5], [9], [12], as antioxidants are related with inhibition of mutagenesis [89]. This is further supported by the previously reported development of oxidative stress by MMC [74]. In any case these assumptions are not to be overestimated and further experiments are required to elucidate the mechanism by which CMW exerts its beneficial activity. Since this is the first report of anti-genotoxic activities of the CMW, further confirmation of these results could contribute to the development of herbal remedies containing natural active principles capable of compensating DNA damage and its subsequent outcomes such as cancer, accelerated ageing or degenerative conditions [90]–[93].

In conclusion, our work provides novel evidence that CMW does not exhibit any genotoxic or recombinogenic activity under our *in vitro* and *in vivo* experimental conditions. Moreover, CMW was found to possess antigenotoxic activity against the alkylating mutagen, MMC. The absence of the genotoxicity and the promising antigenotoxic activity of CMW suggest that this extract may contain phytopharmaceutical molecules of interest that could be used in a range of prospective applications in human healthcare. Although these results highlight the potential antigenotoxic properties of CMW, further studies are needed to delineate its pharmacological properties and its potential usefulness as a natural nontoxic dietary product.

## References

[1] Mills JS, White R (1977) Natural resins of art and archaeology: their sources, chemistry and identification. Stud Conserv 22: 12–31.

[2] Margaris NS (1981) Adaptative strategies in plants dominating Mediterranean-type ecosystems. In: di Castri R, Goodall DW, Specht RI, editors. Ecosystems of the world, Mediterranean type Shrublands. New York: Elsevier Science. 309–315.

[3] Serpico M (2000) Resins, amber and bitumen in ancient Egyptian materials and technology. Cambridge: Cambridge University Press. 430 p.

[4] Doukas C (2003) Cosmetics that contain mastic gum and mastic oil. Chem Chron 12: 36–39.

[5] Triantafyllou A, Bikineyeva A, Dikalova A, Nazarewicz R, Lerakis S, et al (2011) Anti-inflammatory activity of Chios mastic gum is associated with inhibition of TNF-alpha induced oxidative stress. Nutr J 10: 64. 21645369 10.1186/1475-2891-10-64 PMC3127998

[6] Tassou CC, Nychas GCE (1995) Antimicrobial activity of the essential oil of mastic gum (*P. lentiscus* var. *chia*) on Gram Positive and Gram Negative bacteria in broth and in model food system. Int Biodeter Biodegr 36: 411–420.

[7] Iauk L, Ragusa A, Rapisarda A, Franco S, Nicolosi V (1996) *In vitro* antimicrobial activity of *Pistacia lentiscus* L. extracts: preliminary report. J Chemother 8: 207–209. 8808717 10.1179/joc.1996.8.3.207

[8] Magiatis P, Melliou E, Skaltsounis A, Chinou I, Mitaku S (1999) Chemical composition and antimicrobial activity of the essential oils of *Pistacia lentiscus* var. *chia* . Planta Med 65: 749–752. 10630120 10.1055/s-2006-960856

[9] Assimopoulou AN, Zlatanos SN, Papageorgiou VP (2005) Antioxidant activity of natural resins and bioactive triterpenes in oil substrates. Food Chem 92: 721–727.

[10] Koutsoudaki C, Krsek M, Rodger A (2005) Chemical composition and antibacterial activity of the essential oil and the gum of *Pistacia lentiscus* var. *chia* . J Agric Food Chem 53: 7681–7685. 16190616 10.1021/jf050639s

[11] Zhou L, Satoh K, Takahashi K, Watanabe S, Nakamura W, et al (2009) Reevaluation of anti-inflammatory activity of mastic using activated macrophages. In Vivo 23: 583–589. 19567394

[12] Mahmoudi M, Ebrahimzadeh MA, Nabavi SF, Hafezi S, Nabavi SM, et al (2010) Antiinflammatory and antioxidant activities of gum mastic. Eur Rev Med Pharmacol Sci 14: 765–769. 21061835

[13] Paraschos S, Mitakou S (2012) Skaltsounis (2012) AL Chios gum mastic: a review of its biological activities. Curr Med Chem 19: 2292–2302. 22414110 10.2174/092986712800229014

[14] Al-Habbal MJ, Al-Habbal Z, Huwez F (1984) A double-blind controlled clinical trial of mastic and placebo in the treatment of duodenal ulcer. J Clin Exp Pharmacol Physiol 11: 541–544. 10.1111/j.1440-1681.1984.tb00864.x 6395994

[15] Al-Said M, Ageel AM, Parmar NS, Tariq M (1986) Evaluation of mastic, a crude drug obtained from *Pistacia lentiscus* for gastric and duodenal anti-ulcer activity. J Ethnopharmacol 15: 271–278. 3724207 10.1016/0378-8741(86)90165-0

[16] Takahashi K, Fukazawa M, Motohia H, Ochiai K, Nishikawa H, et al (2003) A pilot study on antiplaque effects of mastic chewing gum in the oral cavity. J Periodontol 74: 501–505. 12747455 10.1902/jop.2003.74.4.501

[17] Dedoussis GV, Kaliora AC, Psarras S, Chiou A, Mylona A, et al (2004) Antiatherogenic effect of *Pistacia lentiscus* via GSH restoration and downregulation of CD36 mRNA expression. Atherosclerosis 174: 293–303. 15136059 10.1016/j.atherosclerosis.2004.02.011

[18] Kaliora AC, Stathopoulou MG, Triantafillidis JK, Dedoussis GV, Andrikopoulos NK (2007) Alterations in the function of circulating mononuclear cells derived from patients with Crohn’s disease treated with mastic. World J Gastroenterol 7: 6031–6036. 10.3748/wjg.v13.45.6031 PMC4250886 18023095

[19] Triantafyllou A, Chaviaras N, Sergentanis TN, Protopapa E, Tsaknis J (2007) Chios mastic gum modulates serum biochemical parameters in a human population. J Ethnopharmacol 111: 43–49. 17150319 10.1016/j.jep.2006.10.031

[20] Dabos KJ, Sfika E, Blatta LJ, Frantzi D, Amygdalos GI, et al (2010) Is Chios mastic gum effective in the treatment of functional dyspepsia? A prospective randomised double-blind placebo controlled trial. J Ethnopharmacol 127: 205–209. 19961914 10.1016/j.jep.2009.11.021

[21] Vallianou I, Peroulis N, Pantazis P, Hatzopoulou-Cladaras M (2011) Camphene, a plant-derived monoterpene, reduces plasma cholesterol and triglycerides in hyperlipidemic rats independently of HMG-CoA reductase activity. Plos One 6: 1–11. 10.1371/journal.pone.0020516 PMC3207810 22073134

[22] Loutrari H, Magkouta S, Pyriochou A, Koika V, Kolisis FN, et al (2006) Mastic oil from *Pistacia lentiscus* var. *chia* inhibits growth and survival of human K562 leukemia cells and attenuates angiogenesis. Nutr Cancer 55: 86–93. 16965245 10.1207/s15327914nc5501_11

[23] He ML, Yuan HK, Jiang AL, Gong AY, Chen WW, et al (2006) Gum mastic inhibits the expression and function of the androgen receptor in prostate cancer cells. Cancer 106: 2547–2555. 16691616 10.1002/cncr.21935

[24] He ML, Li A, Xu CS, Wang SL, Zhang MJ, et al (2007) Mechanisms of antiprostate cancer by gum mastic: NF-kappaB signal as target. Acta Pharmacol Sin 28: 446–452. 17303010 10.1111/j.1745-7254.2007.00536.x

[25] Balan KV, Prince J, Han Z, Dimas K, Cladaras M, et al (2007) Antiproliferative activity and induction of apoptosis in human colon cancer cells treated *in vitro* with constituents of a product derived from *Pistacia lentiscus* L. var. *chia* . Phytomedicine 14: 263–272. 16713222 10.1016/j.phymed.2006.03.009

[26] Magkouta S, Stathopoulos GT, Psallidas I, Papapetropoulos A, Kolisis FN, et al (2009) Protective effects of mastic oil from *Pistacia lentiscus* variation *chia* against experimental growth of lewis lung carcinoma. Nutr Cancer 61: 640–648. 19838938 10.1080/01635580902825647

[27] Huang XY, Wang HC, Yuan Z, Li A, He ML, et al (2010) Gemcitabine combined with gum mastic causes potent growth inhibition and apoptosis of pancreatic cancer cells. Acta Pharmacol Sin 31: 741–745. 20523344 10.1038/aps.2010.54 PMC4002976

[28] Giaginis C, Theocharis S (2011) Current evidence on the anticancer potential of Chios mastic gum. Nutr Cancer 63: 1174–1184. 22044444 10.1080/01635581.2011.607546

[29] Paraschos S, Magiatis P, Gousia P, Economou V, Sakkas H, et al (2011) Chemical investigation and antimicrobial properties of mastic water and its major constituents. Food Chem 129: 907–911. 25212317 10.1016/j.foodchem.2011.05.043

[30] OECD website. Available: http://www.oecd.org/env/ehs/testing/TG487%20Oct%202012%20updated%2029oct.pdf. Accessed 2013 May 31.

[31] Kirsch-Volders M, Decordier I, Elhajouji A, Plas G, Aardema MJ, et al (2011) *In vitro* genotoxicity testing using the micronucleus assay in cell lines, human lymphocytes and 3D human skin models. Mutagenesis 26: 177–184. 21164200 10.1093/mutage/geq068

[32] Graf U, Würgler FE, Katz AJ, Frei H, Juon H, et al (1984) Somatic mutation and recombination test in *Drosophila melanogaster* . Environ Mutagen 6: 153–188. 6423380 10.1002/em.2860060206

[33] Graf U, Abraham SK, Guzman-Rincon J, Würler FE (1998) Antigenotoxicity studies in *Drosophila melanogaster* . Mutat Res 402: 203–209. 9729134 10.1016/s0027-5107(97)00298-4

[34] Banfi S, Borsani G, Rossi E, Bernard L, Guffanti A, et al (1996) Identification and mapping of human cDNAs homologous to *Drosophila* mutant genes through EST database searching. Nat Genet 13: 167–174. 8640222 10.1038/ng0696-167

[35] Reiter LT, Potocki L, Chien S, Gribskov M, Bier E (2001) A Systematic analysis of human disease-associated gene sequences in *Drosophila melanogaster* . Genome Res 11: 1114–1125. 11381037 10.1101/gr.169101 PMC311089

[36] Apidianakis Y, Rahme LG (2011) *Drosophila melanogaster* as a model for human intestinal infection and pathology. Dis Model Mech 4: 21–30. 21183483 10.1242/dmm.003970 PMC3014343

[37] Kim SI, Jung JW, Ahn YJ, Restifo LL, Kwon HK (2011) *Drosophila* as a model system for studying lifespan and neuroprotective activities of plant-derived compounds. J Asia-Pac Entomol 14: 509–517.

[38] Kounatidis I, Ligoxygakis P (2012) *Drosophila* as a model system to unravel the layers of innate immunity to infection. Open Biol 2: 120075. 22724070 10.1098/rsob.120075 PMC3376734

[39] Bonassi S, El-Zein R, Bolognesi C, Fenech M (2011) Micronuclei frequency in peripheral blood lymphocytes and cancer risk: evidence from human studies. Mutagenesis 26: 93–100. 21164188 10.1093/mutage/geq075

[40] Sengstag C (1994) The role of mitotic recombination in carcinogenesis. Crit Rev Toxicol 24: 323–353. 7857521 10.3109/10408449409017922

[41] Clare G, Lorenzon G, Akhurst L, Marzin D, van Delft J, et al (2006) SFTG International collaborative study on the *in vitro* micronucleus test. II. Using human lymphocytes. Mutat Res 607: 37–60. 16765631 10.1016/j.mrgentox.2006.04.001

[42] Surrallés J, Carbonell E, Marcos R, Degrassi F, Antoccia A, et al (1992) A collaborative study on the improvement of the micronucleus test in cultured human lymphocytes. Mutagenesis 7: 407–410. 1474915 10.1093/mutage/7.6.407

[43] Vlastos D, Stephanou G (1998) Effects of cetirizine dihydrochloride on human lymphocytes *in vitro*: micronucleus induction. Evaluation of clastogenic and aneugenic potential using CREST and FISH assays. Arch Dermatol Res 290: 312–318. 9705162 10.1007/s004030050310

[44] Papapaulou P, Vlastos D, Stephanou G, Demopoulos NA (2001) Linuron cytogenetic activity on human lymphocutes treated *in vitro*. Evaluation of clastogenic and aneugenic potential using Cytokinesis Block Micronucleus Assay in combination with Fluorescence in situ Hybridization (FISH). Fresen Environ Bull 10: 421–437.

[45] Demsia G, Vlastos D, Goumenou M, Matthopoulos DP (2007) Assessment of the genotoxicity of imidacloprid and metalaxyl in cultured human lymphocytes and rat bone marrow. Mutat Res 634: 32–39. 17950659 10.1016/j.mrgentox.2007.05.018

[46] Fenech M (1997) The advantages and disadvantages of the cytokinesis-block micronucleus method. Mutat Res 392: 11–18. 9269327 10.1016/s0165-1218(97)00041-4

[47] Fenech M, Chang WP, Kirsch-Volders M, Holland N, Bonassi S, et al (2003) HUMN project: detailed description of the scoring criteria for the cytokinesis-block micronucleus assay using isolated human lymphocyte cultures. Mutat Res 534: 65–75. 12504755 10.1016/s1383-5718(02)00249-8

[48] Surrallés J, Xamena N, Creus A, Catalan J, Norppa H, et al (1995) Induction of micronuclei by five pyrethroid insecticides in whole-blood and isolated human lymphocyte cultures. Mutat Res 341: 169–184. 7529358 10.1016/0165-1218(95)90007-1

[49] Lindsley DL, Zimm GG (1992) The genome of *Drosophila melanogaster*. San Diego: Academic Press. 1133 p.

[50] Marec F, Gelbic I (1994) High recombinagenic activities of three antiviral agents adenine derivatives, in the *Drosophila* wing spot test. Mutat Res 311: 305–317. 7526196 10.1016/0027-5107(94)90189-9

[51] Graf U, van Schaik N (1992) Improved high bioactivation cross for the wing somatic mutation and recombination test in *Drosophila melanogaster* . Mutat Res 271: 59–67. 1371830 10.1016/0165-1161(92)90032-h

[52] Selby PB, Olson WH (1981) Methods and criteria for deciding whether specific- locus mutation-rate data in mice indicate a positive, negative, or inconclusive result. Mutat Res 83: 403–418.

[53] Frei H, Würgler FE (1988) Statistical methods to decide whether mutagenicity test data from *Drosophila* assays indicate a positive, negative, or inconclusive result. Mutat Res 203: 297–308. 3136327 10.1016/0165-1161(88)90019-2

[54] Kastenbaum MA, Bowman KO (1970) Tables for determining the statistical significance of mutation frequencies. Mutat Res 9: 527–549. 5424720 10.1016/0027-5107(70)90038-2

[55] Margolin BH, Collings BJ, Mason JM (1983) Statistical analysis and sample-size determinations for mutagenicity experiments with binomial responses. Environ Mutagen 5: 705–716. 6617600 10.1002/em.2860050509

[56] Frei H, Würgler FE (1995) Optimal experimental design and sample size for the statistical evaluation of data from somatic mutation and recombination tests (SMART) in *Drosophila* . Mutat Res 334: 247–258. 7885379 10.1016/0165-1161(95)90018-7

[57] Abraham SK (1994) Antigenotoxicity of coffee in the *Drosophila* assay for somatic mutation and recombination. Mutagenesis 9: 383–386. 7968582 10.1093/mutage/9.4.383

[58] Kang JS, Wanibuchi H, Salim EI, Kinoshita A, Fukushima S (2007) Evaluation of the toxicity of mastic gum with 13 weeks dietary administration to F344 rats. Food Chem Toxicol 45: 494–501. 17092621 10.1016/j.fct.2006.09.013

[59] Loizzo MR, Tundis R, Menichini F, Saab AM, Statti GA, et al (2008) Antiproliferative effects of essential oils and their major constituents in human renal adenocarcinoma and amelanotic melanoma cells. Cell Proliferat 41: 1002–1012. 10.1111/j.1365-2184.2008.00561.x PMC6496814 19040575

[60] Garozzo A, Timpanaro R, Bisignano B, Furneri PM, Bisignano G, et al (2009) In vitro antiviral activity of *Melaleuca alternifolia* essential oil. Lett Appl Microbiol 49: 806–808. 19843207 10.1111/j.1472-765X.2009.02740.x

[61] Hortháthová E, Slameňová D, Maršálková L, Šramková M, Wsólová L (2009) Effects of borneol on the level of DNA damage induced in primary rat hepatocytes and testicular cells by hydrogen peroxide. Food Chem Toxicol 47: 1318–1323. 19285536 10.1016/j.fct.2009.03.002

[62] Kim HJ, Neophytou K (2009) Natural anti-Inflammatory compounds for the management and adjuvant therapy of inflammatory bowel disease and its drug delivery system. Arch Pharm Res 32: 997–1004. 19641880 10.1007/s12272-009-1704-1

[63] Slameňová D, Hortháthová E, Wsólová L, Šramková M, Navarová J (2009) Investigation of anti-oxidative, cytotoxic, DNA-damaging and DNA-protective effects of plant volatiles eugenol and borneol in human-derived HepG2, Caco-2 and VH10 cell lines. Mutat Res 677: 46–52. 19501671 10.1016/j.mrgentox.2009.05.016

[64] Nibret E, Wink M (2010) Trypanocidal and antileukaemic effects of the essential oils of *Hagenia abyssinica*, *Leonotis ocymifolia*, *Moringa stenopetala*, and their main individual constituents. Phytomedicine 17: 911–920. 20359874 10.1016/j.phymed.2010.02.009

[65] Mademtzoglou D, Akmoutsou P, Kounatidis I, Franzios G, Drosopoulou E, et al (2011) Applying the *Drosophila* wing spot test to assess the genotoxic impact of 10 essential oil constituents used as flavouring agents or cosmetic ingredients. Flavour Fragr J 26: 447–451.

[66] Bestmann HJ, Classen B, Vostrowsky O, Klingauf F, Stein U (1988) Botanical insecticides, VIII: the synergistic effect of carvone and pyrethrin I in the essential oil of *Chrysanthemum balsamita* L. J Appl Entomol. 106: 144–149.

[67] Franzios G, Mirotsou M, Hatziapostolou E, Kral J, Scouras ZG, et al (1997) Insecticidal and genotoxic activities of mint essential oils. J Agric Food Chem 45: 2690–2694.

[68] Abdelwahed A, Bouhlel I, Skandrani I, Valenti K, Kadri M, et al (2007) Study of antimutagenic and antioxidant activities of gallic acid and 1,2,3,4,6-pentagalloylglucose from *Pistacia lentiscus*. Confirmation by microarray expression profiling. Chem Biol Interact 165: 1–13. 17129579 10.1016/j.cbi.2006.10.003

[69] Abdelwahed A, Bhouri W, Neffati A, Ben Sghaier M, Boubaker J, et al (2009) Antigenotoxic and antioxidant activities of fruit extracts from (Tunisian) *Pistacia lentiscus* . Food Sci Tech Int 15: 215–222.

[70] Abraham SK, Singh SP (1999) Anti-genotoxicity and glutathione S-transferase activity in mice pretreated with caffeinated and decaffeinated coffee. Food Chem Toxicol 37: 733–739. 10496374 10.1016/s0278-6915(99)00053-8

[71] Lehmann M, Graf U, Reguly ML, Rodrigues De Andrade HH (2000) Interference of tannic acid on the genotoxicity of mitomycin C, methylmethanesulfonate, and nitrogen mustard in somatic cells of *Drosophila melanogaster* . Environ Mol Mutagen 36: 195–200. 11044900 10.1002/1098-2280(2000)36:3<195::aid-em2>3.0.co;2-b

[72] Niikawa M, Shin S, Nagase H (2007) Suppressive effect of post- or pre-treatment of aspirin metabolite on mitomycin C-induced genotoxicity using the somatic mutation and recombination test in *Drosophila melanogaster* . Biomed Pharmacother 61: 113–119. 17275250 10.1016/j.biopha.2006.07.094

[73] Speit G, Linsenmeyer R, Schütz P, Kuehner S (2012) Insensitivity of the *in vitro* cytokinesis-block micronucleus assay with human lymphocytes for the detection of DNA damage present at the start of the cell culture. Mutagenesis 27: 743–747. 22869611 10.1093/mutage/ges041

[74] Turkez H, Aydin E, Aslan A (2012) *Xanthoria elegans* (Link) (lichen) extract counteracts DNA damage and oxidative stress of mitomycin C in human lymphocytes. Cytotechnology 64: 679–686. 22447390 10.1007/s10616-012-9447-0 PMC3488371

[75] Lorge E, Thybaud V, Aardema MJ, Oliver J, Wakata A, et al (2006) SFTG International collaborative study on the *in vitro* micronucleus test. I. General conditions and overall conclusions of the study. Mutat Res 607: 13–36. 16815079 10.1016/j.mrgentox.2006.04.006

[76] Al-Zubairi AS, Abdul AB, Syam MM (2010) Evaluation of the genotoxicity of zerumbone in cultured human peripheral blood lymphocytes. Toxicol In Vitro 24: 707–712. 20123012 10.1016/j.tiv.2010.01.011

[77] Mazumdar M, Giri S, Giri A (2011) Role of quercetin on mitomycin C induced genotoxicity: analysis of micronucleus and chromosome aberrations *in vivo* . Mutat Res 721: 147–152. 21256974 10.1016/j.mrgentox.2011.01.007

[78] Doi K, Wei M, Kitano M, Uematsu N, Inoue M, et al (2009) Enhancement of preneoplastic lesion yield by Chios Mastic Gum in a rat liver medium-term carcinogenesis bioassay. Toxicol Appl Pharm 234: 135–142. 10.1016/j.taap.2008.10.001 18977376

[79] Kaya B, Creus A, Velázquez A, Yanikoglu A, Marcos R (2002) Genotoxicity is modulated by ascorbic acid. Studies using the wing spot test in *Drosophila* . Mutat Res 520: 93–101. 12297148 10.1016/s1383-5718(02)00173-0

[80] De Rezende AAA, e Silva MLA, Tavares DC, Cunha WR, Rezende KCS, et al (2011) The effect of the dibenzylbutyrolactolic lignan (-)-cubebin on doxorubicin mutagenicity and recombinogenicity in wing somatic cells of *Drosophila melanogaster* . Food Chem Toxicol 49: 1235–1241. 21385598 10.1016/j.fct.2011.03.001

[81] Resende FA, Tomazella IM, Barbosa LC, Ponce M, Furtado RA, et al (2011) Effect of the dibenzylbutyrolactone lignan (−)-hinokinin on doxorubicin and methyl methanesulfonate clastogenicity in V79 Chinese hamster lung fibroblasts. Mutat Res 700: 62–66. 10.1016/j.mrgentox.2010.04.023 20452459

[82] De Rezende AAA, Munari CC, de Oliveira PF, Ferreira NH, Tavares DC, et al (2013) A comparative study of the modulatory effects of (-)-cubebin on the mutagenicity/recombinogenicity induced by different chemical agents. Food Chem Toxicol 55: 645–652. 23402860 10.1016/j.fct.2013.01.050

[83] Kato FH, Viana NI, Santini CB, de Souza CG, Veneziani RC, et al (2012) Assessment of the *in vitro* and *in vivo* genotoxic and antigenotoxic effects of pimaradienoic acid in mammalian cells. Mutat Res 749: 87–92. 22985806 10.1016/j.mrgentox.2012.09.001

[84] Mitić-Ćulafić D, Žegura B, Nikolić B, Vuković-Gačić B, Knežević-Vukčević J, et al (2009) Protective effect of linalool, myrcene and eucalyptol against t-butyl hydroperoxide induced genotoxicity in bacteria and cultured human cells. Food Chem Toxicol 47: 260–266. 19049815 10.1016/j.fct.2008.11.015

[85] Chan NLS, Wang H, Wang Y, Leung HY, Leung LK (2006) Polycyclic aromatic hydrocarbon-induced CYP1B1 activity is suppressed by perillyl alcohol in MCF-7 cells. Toxicol Appl Pharm 213: 98–104. 10.1016/j.taap.2005.10.002 16307765

[86] De Flora S, Ramel C (1988) Mechanisms of inhibitors of mutagenesis and carcinogénesis. Classification and overview. Mutat Res 202: 285–306. 3057362 10.1016/0027-5107(88)90193-5

[87] Hodnick WF, Sartorelli AC (1993) Reductive activation of mitomycin C by NADH: cytochrome b5 reductase. Cancer Res 53: 4907–4491. 8402680

[88] Tomasz M, Palom Y (1997) The mitomycin bioreductive antitumor agents: cross-linking and alkylation of DNA as the molecular basis of their activity. Pharmacol Ther 76: 73–87. 9535170 10.1016/s0163-7258(97)00088-0

[89] Bakkali F, Averbeck S, Averbeck D, Idaomar M (2008) Biological effects of essential oils–A review. Food Chem Toxicol 46: 446–475. 17996351 10.1016/j.fct.2007.09.106

[90] De Flora S, Izzotti A (2007) Mutagenesis and cardiovascular diseases: molecular mechanisms, risk factors, and protective factors. Mutat Res 621: 5–17. 17383689 10.1016/j.mrfmmm.2006.12.008

[91] Jones S, Chen WD, Parmigiani G, Diehl F, Beerenwinkel N, et al (2008) Comparative lesion sequencing provides insight into tumor evolution. Proc Natl Acad Sci USA 105: 4283–4288. 18337506 10.1073/pnas.0712345105 PMC2393770

[92] Erickson RP (2010) Somatic gene mutation and human disease other than cancer: an update. Mutat Res 705: 96–106. 20399892 10.1016/j.mrrev.2010.04.002

[93] Frank SA (2010) Evolution in health and medicine Sackler colloquium: Somatic evolutionary genomics: mutations during development cause highly variable genetic mosaicism with risk of cancer and neurodegeneration. Proc Natl Acad Sci USA 107 Suppl 11725–1730. 19805033 10.1073/pnas.0909343106 PMC2868288

